# Essential Oil Enriched with Oxygenated Constituents from Invasive Plant *Argemone ochroleuca* Exhibited Potent Phytotoxic Effects

**DOI:** 10.3390/plants9080998

**Published:** 2020-08-05

**Authors:** Ahmed M. Abd-ElGawad, Abd El-Nasser G. El Gendy, Abdulaziz M. Assaeed, Saud L. Al-Rowaily, Elsayed A. Omer, Basharat A. Dar, Wafa’a A. Al-Taisan, Abdelsamed I. Elshamy

**Affiliations:** 1 Plant Production Department, College of Food and Agriculture Sciences, King Saud University, P.O. Box 2460, RIYADH 11451, Saudi Arabia; assaeed@ksu.edu.sa (A.M.A.); srowaily@ksu.edu.sa (S.L.A.-R.); baseratali@gmail.com (B.A.D.); 2 Department of Botany, Faculty of Science, Mansoura University, Mansoura 35516, Egypt; 3 Medicinal and Aromatic Plants Research Department, National Research Centre, 33 El Bohouth St., Dokki, Giza 12622, Egypt; aggundy_5@yahoo.com (A.E.-N.G.E.G.); sayedomer2001@yahoo.com (E.A.O.); 4 Department of Biology, College of Science, Imam Abdulrahman Bin Faisal University, P.O. Box 1982, Dammam 31441, Saudi Arabia; Waltaisan@iau.edu.sa; 5 Chemistry of Natural Compounds Department, National Research Centre, 33 El Bohouth St., Dokki, Giza 12622, Egypt; elshamynrc@yahoo.com

**Keywords:** *Argemone ochroleuca*, essential oil, invasive plants, oxygenated terpenes, allelopathy

## Abstract

Invasive species are considered as one of the major threats to ecosystems worldwide. Although invasive plants are regarded as a foe, they could be considered as natural resources for valuable bioactive compounds. The present study aimed to characterize the chemical composition of the essential oil (EO) from the invasive plant *Argemone ochroleuca* Sweet, collected from Saudi Arabia, as well as to evaluate its phytotoxic activity. Seventy-four compounds were characterized via GC-MS analysis of EO representing 98.75% of the overall mass. The oxygenated constituents (79.01%) were found as the main constituents, including mono- (43.27%), sesqui- (17.67%), and di-terpenes (0.53%), as well as hydrocarbons (16.81%) and carotenoids (0.73%). Additionally, 19.69% from the overall mass was characterized as non-oxygenated compounds with mono- (1.77%), sesquiterpenes (17.41%), and hydrocarbons (0.56%) as minors. From all identified constituents, *trans*-chrysanthenyl acetate (25.71%), *γ*-cadinene (11.70%), oleic acid, methyl ester (7.37%), terpinene-4-ol (4.77%), dihydromyrcenol (2.90%), *α*-muurolene (1.77%), and *γ*-himachalene (1.56%) were found as abundant. The EO of *A. ochroleuca* showed significant phytotoxic activity against the test plant *Lactuca sativa* and the noxious weed *Peganum harmala.* The EO attained IC_50_ values of 92.1, 128.6, and 131.6 µL L^−1^ for seedling root growth, germination, and shoot growth of *L. sativa*, respectively, while it had IC_50_ values of 134.8, 145.7, and 147.9 µL L^−1^, respectively, for *P. harmala*. Therefore, this EO could be used as a bioherbicide against weeds, while further study is recommended for the characterization of the authentic materials of the main compounds in the EO as well as for the evaluation of potency of this oil on a field scale and the determination of its biosafety.

## 1. Introduction

Plants are promising natural resources for essential oils (EOs) with a complex mixture of secondary metabolites, including mono-, sesqui-, and di-terpenoids, in addition to hydrocarbons [1,2]. The chemical compounds of EOs were biosynthesized via the different isoprenoid pathways [3]. The EOs have been described as potent biological agents such as phytotoxic [4,5,6,7], antimicrobial [8], anti-inflammatory, antipyretic [9], antiulcer [10], and hepatoprotective [11]. The bioactivities of EOs are directly correlated and associated with their chemical constituents [5]. Additionally, EOs can be used widely in several industries as controlling agents for various harmful microorganisms that cause post-harvest diseases, like phytopathogenic and food-borne organisms [12].

Plants belonging to *Argemone* genus are important medicinal plants [13]. Several traditional uses were described from these plants, such as expectorant, demulcent, diuretic, emetic, and treatment in chronic skin diseases [14]. The oils from *A. mexicana* seeds were widely used in the treatment of several diseases like intestinal infections, ulcers, dysentery, asthma, and hypertension [15,16,17]. Leaves, seeds, and flowers of *A. mexicana* were stated to have numerous medicinal uses, such as coughs, and maintenance of blood cholesterol and normal circulation as well as anti-venom [18,19,20,21]. The chemical characterization of *Argemone* plant species afforded various metabolites, such as terpenoids, alkaloids, phenolics, and flavonoids [14].

In Saudi Arabia, *A. mexicana* and *A. ochroleuca* Sweet. were recorded, while the later was described as an abundant and invasive plant in several habitats such as roadsides and disturbed areas [22]. *A. ochroleuca* has been reported as a toxic plant to herbivores and as noxious and competitor weed for many crops due to its allelopathic effect [23]. Control of weeds in an environmentally friendly way is often considered a challenge in agricultural practices to avoid the harmful effects of synthetic herbicides. Therefore, many studies were devoted to finding alternative products derived from natural sources [24]. The EOs are described as promising natural compounds for controlling weeds [1,4,25].

Up to our knowledge, there are no studies concerning the chemical constituents of *A. ochroleuca* EO. We hypothesized that *A. ochroleuca* is a weed, but it may contain phytotoxic constituents such as EOs that could be used as bioherbicides against other weeds; thus, if EO of *A. ochroleuca* has potent herbicidal activity, it could be a possible way to integrate this oil as bioherbicide and make use of this plant. Therefore, the present work provided for the first time (i) the chemical profile of EO of *A. ochroleuca* as well as (ii) the potential phytotoxic activity of this EO against the test plant *Lactuca sativa* L. and the noxious weed *Peganum harmala* L.

## 2. Results and Discussion

### 2.1. EO Chemical Profile

**Figure 1:**
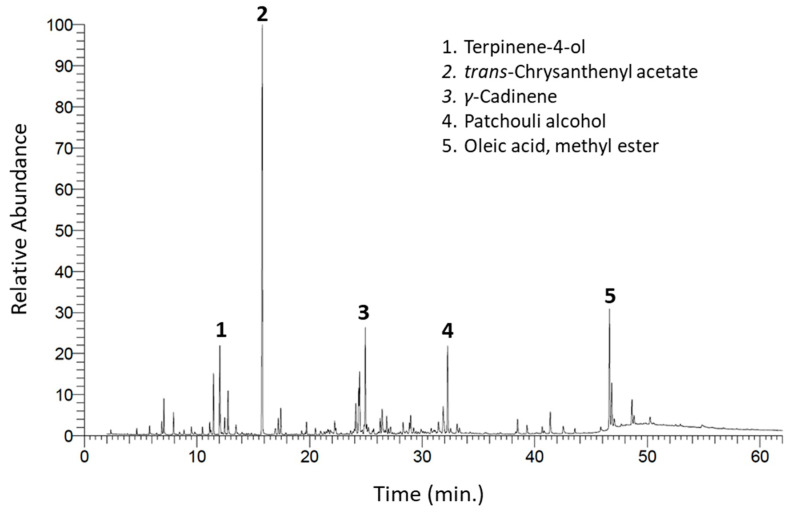
Gas chromatography-mass spectrometry (GC-MS) chromatogram of *Argemone ochroleuca* essential oil (EO). The major compounds’ peaks were numbered 1–5.

The hydrodistillation extraction of above-ground parts of *A. ochroleuca* provided 0.031 ± 0.001% (*v*/*w*) of a colorless oil. The GC-MS analysis of EO was performed, and the chromatogram, including representation of the main constituents over main peaks, is shown in Figure 1. The chemical profile of this plant was described here for the first time. The full chemical profile is presented in Table 1, which is composed of 70 compounds, representing 98.75% of the total mass. Among the overall identified mass, terpenes were found as the main compounds with a concentration of 80.65%, including mono-, sesqui-, and diterpenes.

**Figure 2:**
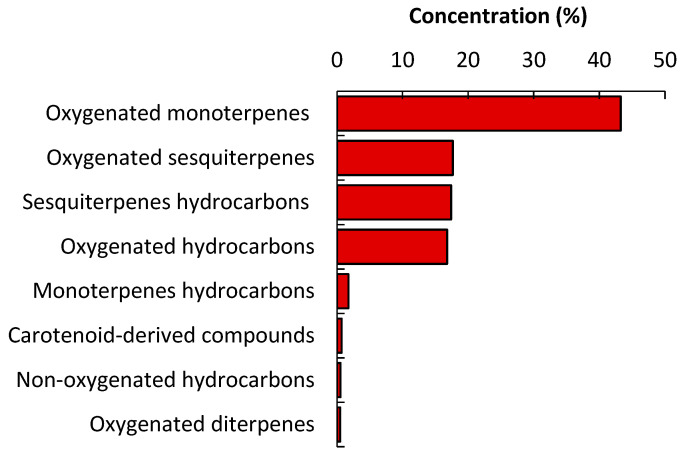
Concentrations of different classes of the compounds in the EO of *Argemone ochroleuca*.

**Figure 3:**
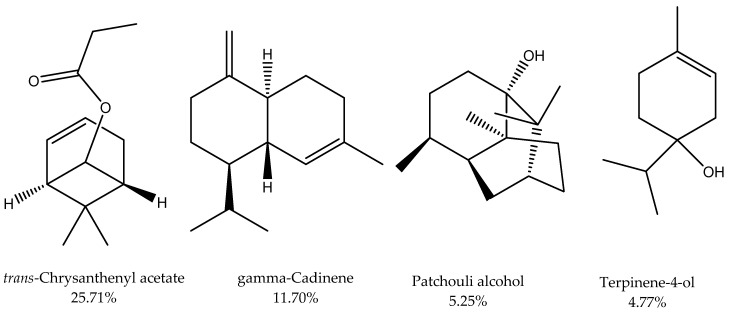
Representative structures of the main compounds.

This result is in harmony with almost all of the described EOs derived from the plant kingdom [26]. The EOs derived from plants were characterized by the abundance of compounds structurally based on isoprene units, especially the terpenoids [3]. Monoterpenes were characterized as the major compounds (Figure 2), representing 45.05%, including oxygenated monoterpenes (43.27%) and monoterpenes hydrocarbons (1.77%). Fifteen monoterpenes in oxygenated forms were found to be the main class of compounds among all the identified constituents with an abundance of *trans*-chrysanthenyl acetate (25.71%, Figure 3), terpinene-4-ol (4.77%, Figure 3), and dihydromyrcenol (2.90%). However, *cis*-*P*-2-menthen-1-ol was represented as the minor compound from all the identified oxygenated compounds. On the other side, three monoterpene hydrocarbons were identified representing 1.77% of the whole mass including m-cymene (0.97%), *γ*-terpinene (0.55%), and *α*-terpinolene (0.25%). The *trans*-Chrysanthenyl acetate, a major monoterpene in our study, was already reported as the main compound in EOs of numerous plant species belonging to different families such as *Artemisia absinthium* [27], *Artemisia herba-alba* [28], *Chrysanthemum coronarium* [29]. *Anthemis maritima* [30], *Tanacetum santolinoides* [31], *Bupleurum montanum*, and *B. plantagineum* [32].

The sesquiterpenes were the second characteristic compounds in EO of *A. ochroleuca,* with a concentration of 35.08% comprising oxygenated (17.67%) and non-oxygenated compounds (17.41%). Out of twenty-three oxygenated sesquiterpenes, patchouli alcohol (5.25%, Figure 3) and agarospirol (2.00%) were identified as main compounds, while isoaromadendrene epoxide was found as a minor one. Furthermore, nine sesquiterpene hydrocarbons were identified, including *γ*-cadinene (11.70%, Figure 3), *α*-muurolene (1.77%), and *γ*-himachalene (1.56%), as majors, while isocaryophillene (0.14%) was identified as a minor one. Patchouli alcohol (Figure 3) has been described as a potent medicinal compound with various activities such as anti-influenza [33], anti-tumor [34], and anti-inflammatory [35]. Rifai and Soekamto [36] described the purification and abundance of patchouli alcohol in EO derived from *Pogostemon cablin*.

Diterpenes were rarely characterized in the EOs derived from the plant kingdom [37]. Phytol, a common oxygenated diterpenoid in EOs of plant species, was the only identified compound in EO of *A. ochroleuca* with a low concentration (0.53%). The identification of this compound in the EO of this plant was in agreement with that reported in *A. mexicana* [38].

Hydrocarbons (17.37%) represented remarkable constituents of the *A. ochroleuca* EO, including oxygenated (16.81%) and non-oxygenated compounds (0.56%) (Figure 2). Among all identified hydrocarbons, 16 oxygenated compounds were characterized, with abundance of oleic acid, methyl ester (7.37%), and gamolenic acid (2.58%), while ethyl oleate (0.14%) was detected as minor compound. Additionally, only two compounds (2-*n*-pentylfuran and *n*-hexadecane) were identified as non-oxygenated hydrocarbons. The carotenoids, including dihydroedulan II and *trans*-*α*-ionone, were characterized in the EO of *A. ochroleuca* with a concentration of 0.49% and 0.24%, respectively. It is pertinent to mention here that carotenoids (tetraterpenoids) are reported as abundant pigments in numerous EOs derived from wild plants, vegetables, and fruits [39].

**Figure 4:**
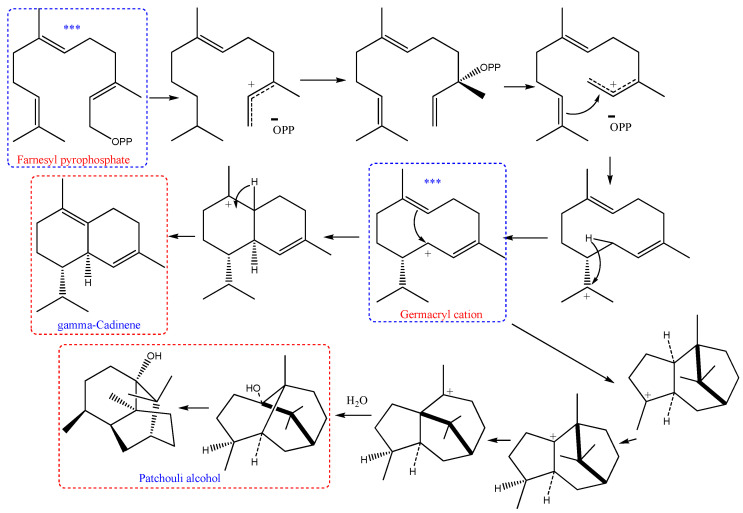
Plausible biosynthetic pathway of gamma-cadinene [40] and patchouli alcohol [41]. *** the common starting and intermediate in biosynthetic of both compounds.

The biosynthetic pathways of the identified compounds might describe the relationship between all the constituents due to the similar starting and/or intermediate compounds as well as the pathway itself. The plausible biosynthetic pathways of the two major compounds, *γ*-cadinene, and patchouli alcohol, as examples, were united at the start with intermediate-compound, farnesyl pyrophosphate that biosynthetically transformed to germacryl cation [40,41] as described in Figure 4. From this brief overview, we can detect the theory that the biosynthetic of the constituents of EOs might establish the relationships between these constituents during the growth of the plant.

### 2.2. Phytotoxic Activity of the A. ochroleuca Essential Oil

**Figure 5:**
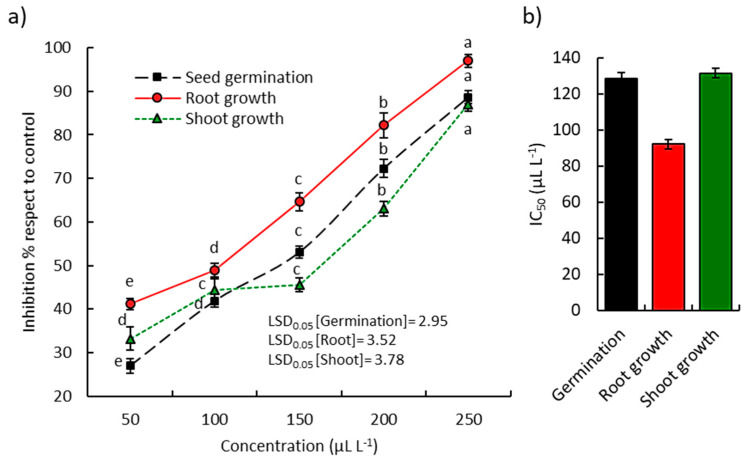
Phytotoxic activity of *Argemone ochroleuca* essential oil on the germination, root, and shoot growth of *Lactuca sativa*. (**a**) Effect of different concentrations and (**b**) the IC_50_ values. Different letters per each line indicate significant differences among treatments at *p* ≤ 0.05 (Tukey’s HSD test).

**Figure 6:**
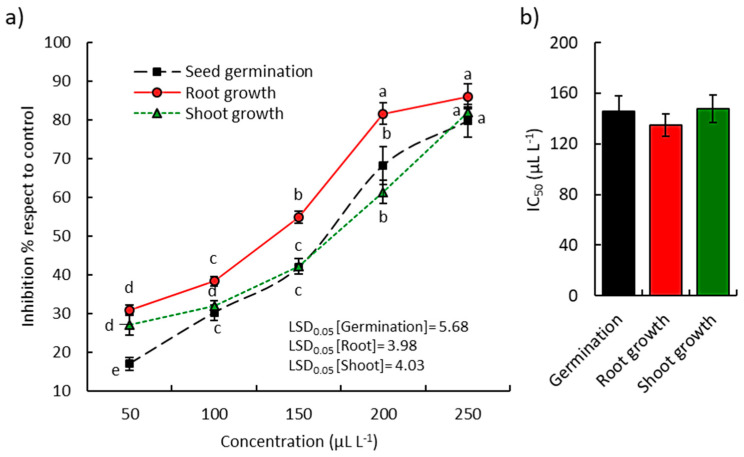
Phytotoxic activity of *Argemone ochroleuca* essential oil on the germination, root, and shoot growth of *Peeganum harmala*. (**a**) Effect of different concentrations and (**b**) the IC_50_ values. Different letters per each line indicate significant differences among treatments at *p* ≤ 0.05 (Tukey’s HSD test).

The EO from the above-ground parts of *A. ochroleuca* showed significant phytotoxic activity on seed germination and seedling development of *L. sativa* (Figure 5a) and *P. harmala* (Figure 6a).

At the highest concentration of the EO (250 µL L^−1^), the seed germination, shoot growth, and root growth of the *L. sativa* seedling were reduced by 88.6, 86.9, and 97.0%, respectively (Figure 5a). According to IC_50_ values, the root was the most inhibited with an IC_50_ value of 92.1 µL L^−1^ (Figure 5b), followed by germination (128.6 µL L^−1^) and finally the shoot (131.6 µL L^−1^). On the other hand, the EO of *A. ochroleuca* at the lowest concentrations (50, 100, and 150 µL L^−1^) revealed significant phytotoxic activity against the germination, shoot growth, and root growth of the noxious weed *P. harmala*, while at the highest concentrations (200 and 250 µL L^−1^) it revealed a sharp increase in the phytotoxicity (Figure 6). Based on the IC_50_, the root of *P. harmala* showed the lowest value (134.8 µL L^−1^), while the seed germination and shoot growth attained IC_50_ values of 145.7 and 147.9 µL L^−1^, respectively (Figure 6).

It is clear that the roots of both *L. sativa* and *P. harmala* seedling were more affected with the EO than shoots, and this could be attributed to the direct contact with the EO as well as the permeability of the root membrane [24,42]. In the present study, the potent phytotoxic activity of the *A. ochroleuca* EO could be ascribed to the presence of high oxygenated compounds, particularly the major compounds such as *trans*-chrysanthenyl acetate, *γ*-cadinene, oleic acid-methyl ester, patchouli alcohol, and terpinene-4-ol. The oxygenated compounds of the EOs were reported to have stronger biological activities than non-oxygenated ones [1,2,43,44]. The bicyclic sesquiterpene *γ*-cadinene was reported to have larvicidal activity against malaria, dengue, and filariasis mosquitoes [45], and it is also reported to have antimicrobial activity [46]. In addition, the EO of *Annona salzmannii* showed potent trypanocidal and antitumor activities due to its high content of *γ*-cadinene [47]. The fatty acid methyl esters have been reported to possess larvicidal activity [48] and antibacterial activity [49].

These compounds may act individually or in synergy as phytotoxic agents (allelochemicals). Compared to other reported EOs, the *A. ochroleuca* EO is more phytotoxic against lettuce (plant model) than the EO of *Teucrium polium* [50], *Eucalyptus grandis* and *E. citriodora* [51], *Acacia cyanophylla* [52], and *Eremanthus erythropappus* [53]. The most abundant compound, *trans*-chrysanthenyl acetate, has been reported as the main compound of other plants’ EO, with phytotoxic activity, such as *Artemisia herba-alba* [28] and *Chrysanthemum coronarium* [29]. In addition, the EO rich in *trans*-chrysanthenyl acetate has been reported to have antimicrobial and antioxidant activities [54,55].

The major compound *γ*-cadinene has been reported as major constituent (18.4%) in the essential oil of *Eupatorium adenophorum,* which showed a phytotoxic activity against *Phalaris minor* and *Triticum aestivum* [56]. Additionally, the EO of *Schinus lentiscifolius* showed a phytotoxic effect on lettuce due to the presence of high content of *γ*-cadinene [57]. Although the other major compounds have not been reported as allelochemicals, they could participate in the phytotoxic effect of the *A. ochroleuca* EO, particularly the patchouli alcohol, and terpinene-4-ol, which are oxygenated terpenes [58].

Several modes of action of EOs as allelochemicals were reported, including the inhibition of permeability, cell division, photosynthesis, respiration, enzyme activities, and genomic materials [59,60]. However, the specific mode(s) of action of the major identified terpene compounds in the present study, either alone or in combinations, needs further investigation.

It is worth mentioning that the weed *P. harmala* is considered a noxious weed in several countries, including Saudi Arabia, where it is widely distributed in the northern regions [61]. It is hard to control and needs powerful herbicides or manual uprooting; no reported biological control methods for the *P. harmala* is available [62]. In this context, the present study revealed the potentiality of *A. ochroleuca* EO to control *P. harmala,* this noxious weed, as an eco-friendly bioherbicide, where this oil showed strong phytotoxicity against this weed.

## 3. Material and Methods

### 3.1. Plant Materials Collection, Identification, and Preparation

The above-ground parts of *A. ochroleuca* were collected from a roadside habitat, Al Assir village, Taif, western Saudi Arabia (21°11′27.2″ N 40°40′05.9″ E). The plant specimen was identified according to Chaudhary [63] by Dr. Abdulaziz Assaeed, Professor of Range Ecology, Department of Plant Production, College of Sciences, King Saud University, Saudi Arabia. A voucher specimen of the collected plant is released in the herbarium of King Saud University, with code: KSU-0160115001. The above-ground parts of the healthy plants were collected in paper bags and transferred to the laboratory. The plant materials were dried in shade at room temperature (28 ± 3 °C) for two weeks (until complete dryness), ground into a fine powder, and packed in a paper bag.

### 3.2. Essential Oil Extraction, GC-MS Analysis, and Constituents’ Identification

The EOs were extracted by hydrodistillation from two samples of *A. ochroleuca* above-ground parts via a Clevenger-type apparatus for three hours. The oil layer was collected, and water was removed by 0.5 g of anhydrous Na_2_SO_4_; they were stored in a dark glass vial at 4 °C till further analysis. The yields of the extracted EOs were calculated via the equation 100× (*V*/*W*), where *V*: volume of extracted EO, and *W*: weight of the plant material used in extraction. The chemical composition of the EO samples was analyzed and identified separately by gas chromatography-mass spectrometry (GC-MS) as described in our previously documented work [2,4].

In brief, GC-MS analysis was carried out at the Department of Medicinal and Aromatic Plants Research, National Research Center, Giza, Egypt, using the GC-MS instrument which has TRACE GC Ultra Gas Chromatographs (THERMO Scientific™. Corporate, Waltham, MA, USA) and Thermo Scientific ISQ™ EC single quadrupole mass spectrometer. The GC-MS system is equipped with a TR-5 MS column with dimensions of 30 m × 0.32 mm i.d., 0.25 µm film thickness. At flow rate of 1.0 mL min^−1^, helium was used as carrier gas with split ratio of 1:10. The temperature program was 60 °C for 1 min, rising by 4.0 °C min^−1^ to 240 °C and held for 1 min. A diluted sample in hexane (1 µL) at a ratio of 1:10 (*v*/*v*) was injected, and the injector and detector were held at 210 °C. Mass spectra were recorded by electron ionization (EI) at 70 eV, using a spectral range of *m*/*z* 40–450. The identification of the chemical constituents of the EOs was achieved using Automated Mass spectral Deconvolution and Identification (AMDIS) software, Wiley spectral library collection, NIST library database, retention indices relative to *n*-alkanes (C_8_–C_22_), or appraisal of the mass spectrum with authentic standards.

### 3.3. Phytotoxic Bioassay of the A. ochroleuca Essential Oil

The phytotoxicity of the *A. ochroleuca* EO was performed against *L. sativa* L. as a standard test plant, where it is known to be very sensitive to allelochemicals [64] as well as the noxious weed *P. harmala*. In brief, the seeds of lettuce were purchased from the Agriculture Research Center, Cairo, Egypt, while the seeds of *P. harmala* were collected from Taif, southeast Saudi Arabia. Seeds with uniform size and color were carefully chosen and surface-sterilized via sodium hypochlorite (0.3%). Prior to the experiment, the viability of the seed was performed by the germination of seeds in the Petri plate lined with a filter paper (Whatman No. 1) using distilled water at 25 °C with adjusted light conditions of 16/8 h light/dark cycle. The germination percentage was 97.23 ± 0.5% for *L. sativa* and 90.12 ± 1.02% for *P. harmala*.

To assess the phytotoxicity of the EO, various concentrations (50, 100, 150, 200, and 250 µL L^−1^) were prepared by dilution using 1% Tween^®^ 80 (Sigma-Aldrich, Darmstadt, Germany) as an emulsifying agent. In Petri plates, 20 sterilized seeds of either *L. sativa* or *P. harmala* were spread on sterilized filter paper (Whatman No. 1), 4 mL of each concentration or control (Tween^®^ 80) was poured, and the plates were sealed with Parafilm^®^ tape (Sigma, St. Louis, MO, USA). Five plates were prepared per each concentration, and the experiment was repeated three times. A total of 180 plates (2 plants × 6 treatments [5 concentrations + control] × 5 plates as replications × 3 times) were prepared and incubated in a growth chamber at 25 °C with adjusted light conditions of 16 h/8 h light/dark cycle. After 5 days of incubation for *L. sativa* and 7 days for *P. harmala*, the reduction in seed germination, shoot growth, and root growth of the seedlings were calculated based on the following equation:

(1) $$\mathrm{Inhibition}\;\left(\%\right)=100\times \frac{\left(N_{control}/L_{control}-N_{treatment}/L_{treatment}\right)}{N_{treatment}/L_{control}}$$

where *N* is the number of germinated seeds and *L* is the length of seedling root or shoot.

### 3.4. Statistical Analysis

The experiment of bioassay was designed as a completely randomized design and repeated three times with five replications per each treatment. The data of seed germination and seedling growth inhibition were subjected to one-way ANOVA and followed by Duncan’s HSD post hoc test at a probability level of 0.05.

## 4. Conclusions

The chemical composition of *A. ochroleuca* EO was characterized with highly oxygenated constituents (79.01%), including mono-, sesqui-, di-terpenoids, carotenoids, and hydrocarbons. The *trans*-chrysanthenyl acetate, *γ*-cadinene, oleic acid, methyl ester, patchouli alcohol, and terpinene-4-ol were determined as the main compounds. The *A. ochroleuca* EO exhibited significant phytotoxic activity. The high oxygenation of the EOs constituents was deduced to be correlated with the increase of the phytotoxicity. Therefore, the substantial phytotoxicity of *A. ochroleuca* EO could be ascribed to its high content of the oxygenated compounds (78.28%), and thereby it could be used as an eco-friendly bioherbicide. However, further study is recommended for characterization of the main identified compounds, either singular or in combination at field scale level, as well as for the evaluation of their modes of action and biosafety.

## Figures and Tables

**Table 1:** Essential oil constituents of above-ground parts of *Argemone ochroleuca*.

No	Rt ^a^	Conc. (%) ^b^	Compound	KI ^c^		Identification ^d^
				Lit.	Exp.	
**Monoterpenes hydrocarbons**						
1	6.88	0.55 ± 0.02	*γ*-Terpinene	1062	1061	MS, KI
2	7.92	0.97 ± 0.02	*m*-Cymene	1082	1080	MS, KI
3	8.85	0.25 ± 0.01	*α*-Terpinolene	1088	1089	MS, KI
**Oxygenated Monoterpenes**						
4	7.06	1.41 ± 0.04	Eucalyptol	1033	1035	MS, KI
5	8.46	0.14 ± 0.01	*cis*-*P*-2-Menthen-1-ol	1130	1129	MS, KI
6	9.51	0.42 ± 0.02	*cis*-Verbenol	1142	1141	MS, KI
7	10.49	1.57 ± 0.05	Camphor	1149	1151	MS, KI
8	11.14	0.63 ± 0.03	Pinocarvone	1158	1157	MS, KI
9	11.23	0.22 ± 0.02	*endo*-Borneol	1165	1166	MS, KI
10	11.47	2.90 ± 0.06	Dihydromyrcenol	1072	1172	MS, KI
11	12.03	4.77 ± 0.09	Terpinene-4-ol	1177	1178	MS, KI
12	12.46	0.90 ± 0.03	*p*-Menth-1-en-4-ol	1182	1181	MS, KI
13	12.76	2.23 ± 0.06	*α*-Linalool	1085	1186	MS, KI
14	14.01	0.16 ± 0.01	*α*-Terpineol	1189	1189	MS, KI
15	15.80	25.71 ± 0.21	*trans*-Chrysanthenyl acetate	1235	1237	MS, KI
16	17.45	1.45 ± 0.02	*α*-Damascenone	1391	1391	MS, KI
17	20.98	0.27 ± 0.01	*cis*-Jasmone	1394	1395	MS, KI
18	21.85	0.23 ± 0.01	Dihydrojasmone	1400	1402	MS, KI
19	23.65	0.26 ± 0.02	*α*-Terpinyl propionate	1747	1747	MS, KI
**Sesquiterpenes hydrocarbons**						
20	20.52	0.38 ± 0.02	*α*-Cubebene	1351	1353	MS, KI
21	21.30	0.16 ± 0.01	alfa.Copaene alfa.Copaene	1376	1377	MS, KI
22	23.99	0.14 ± 0.01	Isocaryophillene	1413	1411	MS, KI
23	24.69	0.23 ± 0.02	Aromandendrene	1439	1438	MS, KI
24	25.69	0.34 ± 0.03	Dehydroaromadendrene	1466	1464	MS, KI
25	26.28	0.79 ± 0.03	*β*-Cadinene	1473	1472	MS, KI
26	26.45	1.56 ± 0.05	*γ*-Himachalene	1479	1479	MS, KI
27	30.80	0.34 ± 0.02	*γ*-Muurolene	1477	1478	MS, KI
28	24.11	1.77 ± 0.04	*α*-Muurolene	1499	1497	MS, KI
29	24.95	11.70 ± 0.08	*γ*-Cadinene	1513	1512	MS, KI
**Oxygenated sesquiterpenes**						
30	19.30	0.22 ± 0.02	Nerolidol	1534	1535	MS, KI
31	21.70	0.18 ± 0.01	Davana furan	1399	1398	MS, KI
32	21.62	0.20 ± 0.01	2,6-Di-tert-butyl-4-hydroxy 4-methyl-2,5-cyclohexadien-1-one	1478	1479	MS, KI
33	22.23	0.76 ± 0.02	Davana ether	1483	1483	MS, KI
34	22.34	0.32 ± 0.01	4-epi-cubedol	1494	1496	MS, KI
35	25.23	0.86 ± 0.02	3-methyl-2-butenoic acid, 2,7-dimethyloct-7-en-5-yn-4-yl ester	1521	1523	MS, KI
36	25.60	0.20 ± 0.01	Isolongifolan-8-ol	1531	1531	MS, KI
37	26.85	1.03 ± 0.03	Epiglobulol	1557	1558	MS, KI
38	28.31	0.71 ± 0.02	Ledol	1565	1566	MS, KI
39	28.87	0.63 ± 0.02	Spathulenol	1575	1574	MS, KI
40	28.99	1.11 ± 0.04	Caryophyllene oxide	1581	1583	MS, KI
41	29.24	0.34 ± 0.02	Davanone	1588	1589	MS, KI
42	29.46	0.14 ± 0.01	Isoaromadendrene epoxide	1594	1594	MS, KI
43	29.91	0.34 ± 0.02	salvial-4(14)-en-1-one	1595	1593	MS, KI
44	31.06	0.20 ± 0.01	Widdrol	1597	1598	MS, KI
45	31.16	0.17 ± 0.01	Rosifoliol	1613	1612	MS, KI
46	31.45	0.73 ± 0.02	Fonenol	1627	1625	MS, KI
47	31.87	2.00 ± 0.04	Agarospirol	1646	1647	MS, KI
48	32.26	5.25 ± 0.06	Patchouli alcohol	1659	1661	MS, KI
49	32.53	0.31 ± 0.02	Longifolenaldehyde	1668	1668	MS, KI
50	33.10	0.62 ± 0.02	Juniper camphor	1691	1690	MS, KI
51	33.31	0.33 ± 0.03	Hexahydrofarnesyl acetone	1845	1844	MS, KI
52	38.47	0.87 ± 0.03	*E*, *E*-Farnesyl acetone	1918	1920	MS, KI
53	40.83	0.15 ± 0.01	*α*-Acorenol	2135	2134	MS, KI
**Oxygenated diterpenes**						
54	47.06	0.53 ± 0.03	Phytol	1942	1942	MS, KI
**Non-oxygenated hydrocarbons**						
55	5.79	0.41 ± 0.02	2-*n*-Pentylfuran	993	994	MS, KI
56	29.62	0.15 ± 0.01	*n*-Hexadecane	1600	1600	MS, KI
**Oxygenated hydrocarbons**						
57	2.35	0.16 ± 0.01	*n*-Hexanal	800	801	MS, KI
58	4.66	0.21 ± 0.02	*n*-Nonanal	1098	1097	MS, KI
59	9.81	0.17 ± 0.01	5-Ethyl-3-hepten-2-one	1124	1126	MS, KI
60	27.01	0.29 ± 0.02	2-Methoxy-1,4-benzenediol	1473	1475	MS, KI
61	40.66	0.42 ± 0.02	7,9-Ditertbutyl-1-oxaspiro-(4,5)- deca-6,9-diene-2,8-dione	1775	1775	MS, KI
62	41.38	1.34 ± 0.04	Dibutyl phthalate	1868	1869	MS, KI
63	42.53	0.48 ± 0.02	Palmitic acid, methyl ester	1926	1924	MS, KI
64	43.56	0.32 ± 0.02	Ethyl palmitate	1994	1995	MS, KI
65	45.86	0.39 ± 0.02	Linoleic acid, methyl ester	2092	2090	MS, KI
66	46.64	7.37 ± 0.11	Oleic acid, methyl ester	2108	2110	MS, KI
67	46.84	2.58 ± 0.09	Gamolenic acid	2143	2141	MS, KI
68	47.69	0.14 ± 0.01	Ethyl oleate	2161	2163	MS, KI
69	48.63	1.56 ± 0.05	Oleic acid	2179	2177	MS, KI
70	48.83	0.53 ± 0.02	*n*-Nonadecanoic acid	2236	2237	MS, KI
71	49.85	0.38 ± 0.01	18-Nonadecenoic acid	2256	2256	MS, KI
72	50.24	0.47 ± 0.02	Ethyl-9,12-octadecadienoate	2527	2525	MS, KI
**Carotenoid-derived compounds**						
73	16.98	0.49 ± 0.02	Dihydroedulan II	1284	1284	MS, KI
74	25.08	0.24 ± 0.01	*trans*-*α*-Ionone	1456	1458	MS, KI

^a^ Rt: retention time, ^b^ compound concentration ± standard division; ^c^ KI: published Kovats retention indices (Lit); and experimental Kovats index (Exp.) relative to *n*-alkanes (C_8_–C_28_) (Exp); ^d^ EO constituents identification was performed via comparison of the mass spectral and Kovats indices (KI) with those of NIST Mass Spectral Library (2011) and Wiley Registry of Mass Spectral Data 8th edition and literature.
